# Supplementary material for: Construction of conducting bimetallic organic metal chalcogenides via selective metal metathesis and oxidation transformation
Source: Nat Commun. 2022 Oct 22;13:6294. doi: 10.1038/s41467-022-34118-7 (PMC9588041; doi:10.1038/s41467-022-34118-7)
Supplement: Supplementary file 1 — Supplementary information [file 41467_2022_34118_MOESM1_ESM.pdf]

Supplementary Information

Construction of conducting bimetallic organic metal chalcogenides via selective metal metathesis and oxidation transformation

Jin et al.

Supplementary Note 1. Synthesis and characterizations

Materials

Benzenehexathiol (BHT), Cu_4BHT were prepared according to previous literature[1,2]. Isopropyl alcohol, Ag_2O, ethanol, acetonitrile were purchased from Acros Organics Co., $Ce(NH_4)_2(NO_3)_6$, $AgNO_3$, $Cu(NO_3)_2•3H_2O$ was purchased from Alfa Aesar Co. Ltd., $Cu(CH_3CN)_4BF_4$ was purchased from TCI China. $Ag(CH_3CN)_4BF_4$ was purchased from Sigma Aldrich. All solvents were degassed by the Freeze-Thaw method before using.

Synthesis of Ag_5BHT

Previously, we found that Cu_2O was the ideal reagent for producing highly crystalline Cu_4BHT (ref.[2]). Inspired by this finding, Ag_5BHT with improved crystallinity was prepared from a similar heterogeneous reaction using Ag_2O as the silver source instead of previously used liquid-liquid interface reaction[3]. Under argon atmosphere, 60 mL degassed isopropyl alcohol was added to the mixture of BHT (100 mg, 0.37 mmol) powder and Ag_2O (213 mg, 0.92 mmol) in 100 mL flask. The mixture was sonicated at 0 °C for 3 min, and then heated to 60 °C and stirred for 24 hours to form a dark green powder. During the entire reaction process, tinfoil papers were used to keep away from light due to the light-sensitive Ag_2O. After natural cooling to room temperature, the product was filtered, washed with water, CH_3OH, acetone and diethyl ether in sequence, and then dried at 80 °C under vacuum for 24 h. Yield: 273 mg (92%). Elem. Anal. Calcd. for $Ag_5C_6S_6$: C, 8.97; S, 23.93; Ag, 67.10. Found: C, 9.12; S, 23.32; Ag, 67.43. The composition and crystallinity of the as-prepared sample were firmly confirmed by elemental analysis, powder X-ray diffraction (PXRD), and transmission electron microscopy (TEM) characterizations (Supplementary Figs. 3-5).

Trials for the synthesis of BHT-based bimetallic OMCs

1. The direct synthesis from BHT. Under argon atmosphere, $Cu(NO_3)_2•3H_2O$ (48 mg, 0.2 mmol) and $AgNO_3$ (34 mg, 0.2 mmol) were dissolved in 50 mL degassed ethanol. Then BHT (27 mg, 0.1 mmol) powder was added under constant stirring. The reaction was stirred at room temperature in the dark for 24 h, and a black precipitate was obtained and filtered. The product was subsequently washed with H_2O, ethanol and acetone, and then dried at 80 °C under vacuum for 24 h (No. 1). Other Cu and Ag salts (No. 2-11) were also tested under similar synthetic procedures.
2. Metal metathesis starting from Cu_4BHT with Ag salts. Under argon atmosphere, Cu_4BHT (26 mg, 0.05 mmol) was added to 25 mL degassed ethanol solution of $AgNO_3$ (34 mg, 0.2 mmol). The mixture was heated to 80 °C in the dark and stirred for 24 h. The black precipitate was filtered and washed with H_2O, ethanol and acetone, and then dried at 80 °C under vacuum for 24 h (No. 12). Other conditions (No. 13-15) were also tested under similar synthetic procedures.
3. Metal metathesis starting from Ag_5BHT with Cu(I) salts. Under argon atmosphere, Ag_5BHT (15 mg, 0.019 mmol) was added to 35 mL degassed CH_3OH solution of $Cu(CH_3CN)_4BF_4$ (30 mg, 0.095 mmol). The mixture was heated to 45 °C and stirred for 24 h. The black precipitate was filtered and washed with H_2O, ethanol and acetone, and then dried at 80 °C under vacuum for 24 h (No. 17). Other condition (No. 18) was also tested under similar synthetic procedures.
4. Metal metathesis starting from Ag_5BHT with $Cu(NO_3)_2•3H_2O$. Under argon atmosphere, Ag_5BHT (15 mg, 0.019 mmol) was added to 40 mL degassed ethanol solution of

$Cu(NO_3)_2$•$3H_2O$ (37 mg, 0.152 mmol). The mixture was heated to 80 °C and stirred for 30 h. The black precipitate was filtered and washed with H_2O, ethanol and acetone, and then dried at 80 °C under vacuum for 24 h (No. 16).

Component characterizations. The content of carbon and sulfur were analyzed using Flash EA 1112 (Thermo Fisher Scientific). The metal content was analyzed by inductively coupled plasma-optical emission spectroscopy (ICP-OES, iCAP 6300 Radial, Thermo Scientific). For ICP-OES measurements, the samples were prepared by dissolving them into fuming nitric acid. Then the resulting solution was diluted to a known volume with Milli-Q water. The metal to sulfur ratios were confirmed by EPMA (JEOL, JXA-8100). XPS and UPS were performed using AXIS Ultra-DLD ultrahigh vacuum photoemission spectroscopy system (Kratos Co.). And the pelletized samples were used for measurements. All XPS spectra were calibrated by saturated carbon (C-C) C 1*s* peak at 284.8 eV. Thermogravimetric analysis (TGA) was performed using a TGA Q500 instrument under a nitrogen flow at a heating rate of 10 °C min^{-1}. PXRD patterns were obtained at a PANalytical Empyrean II X-Ray diffractometer using Cu Kα radiation (λ = 1.5406 Å) at 40 kV and 40 mA. Synchrotron powder X-ray (λ = 0.69003 Å) diffraction data collected at the beamline BL14B1 at Shanghai Synchrotron Radiation Facility (SSRF). The samples were measured by capillary transmission mode with the Bragg angle (2θ) from 2° to 40°. Before PXRD measurements, the samples have been well grounded.

Raman spectroscopic characterization. Raman spectra were collected on a LabRAM HR Evolution (HORIBA) Raman spectrometer with the excitation wavelength of 532 nm in the range of 100-2800 cm^{-1} at room temperature.

Supplementary Note 2. Structure determination

The structure of $CuAg_4BHT$ was solved from a combination of rotation electron diffraction (RED) and synchrotron PXRD data. The 3D RED data demonstrates that $CuAg_4BHT$ crystallizes in the monoclinic space group $P2_1/c$ with the unit cell of a = 4.28 Å, b = 8.78 Å, c = 14.51 Å, and β = 94.1°. Based on this set of initial cell parameters, together with the synchrotron PXRD data of $CuAg_4BHT$, the structure was solved by the charge flipping algorithm implemented in the computer program Superflip[4]. The positions of metal atoms (Ag and Cu) and sulfur atoms were located automatically from the electron density map. The benzene rings were added manually according to the chemical structure of BHT.

The structure of $CuAg_2BHT$ was directly determined based on synchrotron PXRD data. The indexing process was performed by EXPO2014 using the program N-TREOR09 (refs.[5,6]), which gave a triclinic unit cell of a = 3.58 Å, b = 8.43 Å, c = 8.80 Å, α = 62.1°, β = 86.1°, γ = 80.8°. The initial cell parameters were refined by le Bail algorithm using Jana2006 (ref.[7]). Background, zero-point, profile shape and asymmetry parameters were refined together to get the profile fitting. The structure was solved with charge flipping algorithm using the computer program Superflip. The final structure models were refined through the Rietveld method using the program Jana2006.

Supplementary Note 3. Computational details

DFT calculation. Geometry optimization and band structure calculations were carried out using VASP package. The PBE exchange-correlation functional[8] with optB88 van der Waals (vdW) dispersion corrections[9] was employed. The plane wave energy cutoff was set to 550 eV. Uniform 2×3×6, 2×3×6, 7×3×3 Monkhorst-Pack k-point meshes were employed for Ag_5BHT, $CuAg_4BHT$ and $CuAg_2BHT$, respectively. The high-symmetry K-points for Ag_5BHT, $CuAg_4BHT$ and $CuAg_2BHT$ are provided in Supplementary Table 8.

Supplementary Note 4. Supplementary figures and tables

Supplementary Table 1. Condition screening for synthesizing a bimetallic OMC via the direct synthesis.

No.	Solvent	Temp (°C)	Cu/Ag salts	Cu/Ag salts/ligand (equiv. / equiv.)	Result
1	ethanol	r.t.	$Cu(NO_3)_2/AgNO_3$	2/2/1	Poor crystallinity
2	ethanol	60	Cu_2O/Ag_2O	1.5/1.5/1	Poor crystallinity
3	ethanol	80	Cu_2O/Ag_2O	1.5/1.5/1	Ag metal
4	methanol	r.t.	$Cu(CH_3CN)_4BF_4/Ag(CH_3CN)_4BF_4$	4/2/1	Poor crystallinity
5	methanol	r.t.	$Cu(CH_3CN)_4BF_4/Ag(CH_3CN)_4BF_4$	2/4/1	Ag metal
6	methanol	r.t.	$Cu(BF_4)_2/AgBF_4$	2/2/1	Poor crystallinity
7	methanol	r.t.	$Cu(OAc)_2/AgOAc$	2/2/1	Poor crystallinity
8	acetonitrile	r.t.	$Cu(CH_3CN)_4BF_4/Ag(CH_3CN)_4BF_4$	2/2/1	Poor crystallinity
9	acetonitrile	50	$Cu(CH_3CN)_4BF_4/Ag(CH_3CN)_4BF_4$	2/2/1	Poor crystallinity
10	ethylene glycol	50	$Cu(NO_3)_2/AgNO_3$	2/2/1	Poor crystallinity
11	H_2O	60	$Cu(OAc)_2/AgOAc$	2/2/1	Poor crystallinity

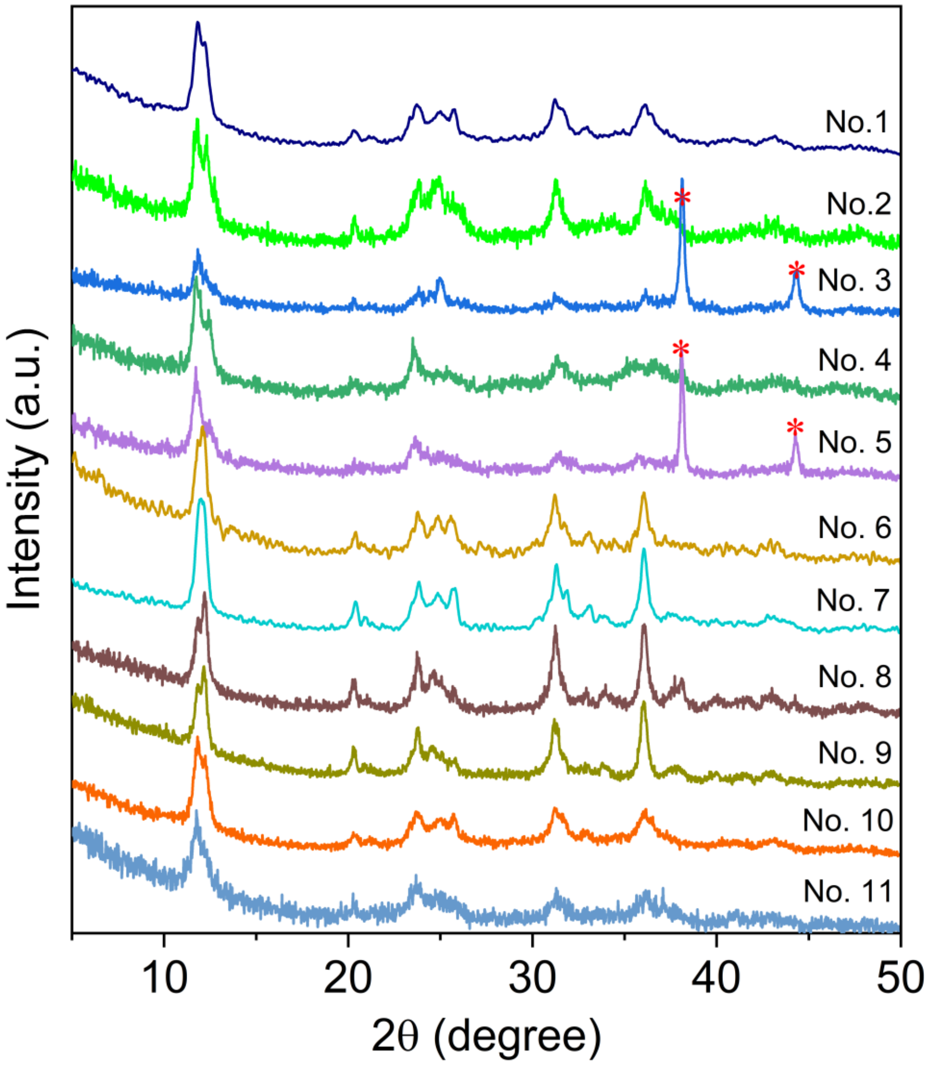

Supplementary Figure 1. PXRD patterns of the products synthesized via the direct synthesis. Due to different reaction rates of BHT and two metal ions, one-pot conditions (Supplementary Table 1) result in unsatisfactory crystallization control. Two typical diffraction peaks at $2\theta = 38.1°$ and 44.3° (marked by the asterisks) can be assigned to (111) and (200) reflections of the Ag FCC phase[10]. Source data are provided as a Source Data file.

Supplementary Table 2. Condition screening for synthesizing a bimetallic OMC from different precursors (Cu_4BHT and Ag_5BHT) via metal metathesis.

No.	Precursor	Temp (°C)	Reaction time (h)	Metal salt	Metal salt/precursor (equiv. / equiv.)	Result
12	Cu_4BHT	80	24	$AgNO_3$	4/1	Ag metal
13	Cu_4BHT	45	24	$Ag(CH_3CN)_4BF_4$	4/1	Ag metal
14	Cu_4BHT	45	12	$AgNO_3$	2/1	Ag metal
15	Cu_4BHT	r.t.	24	$Ag(CH_3CN)_4BF_4$	2/1	Ag metal
16	Ag_5BHT	80	24	$Cu(NO_3)_2$	8/1	Good crystallinity
17	Ag_5BHT	45	24	$Cu(CH_3CN)_4BF_4$	5/1	Ag metal
18	Ag_5BHT	r.t.	24	$Cu(CH_3CN)_4BF_4$	2/1	Ag metal
19	Ag_5BHT	100	7d	$Cu(NO_3)_2$	20/1	Not optimized
20	Ag_5BHT	120	3d	$Mn(NO_3)_2$	5/1	No reaction
21	Ag_5BHT	120	3d	$Fe(NO_3)_2$	5/1	No reaction
22	Ag_5BHT	120	3d	$Co(NO_3)_2$	5/1	No reaction
23	Ag_5BHT	80	24	$Pd(NO_3)_2$	5/1	Pd metal

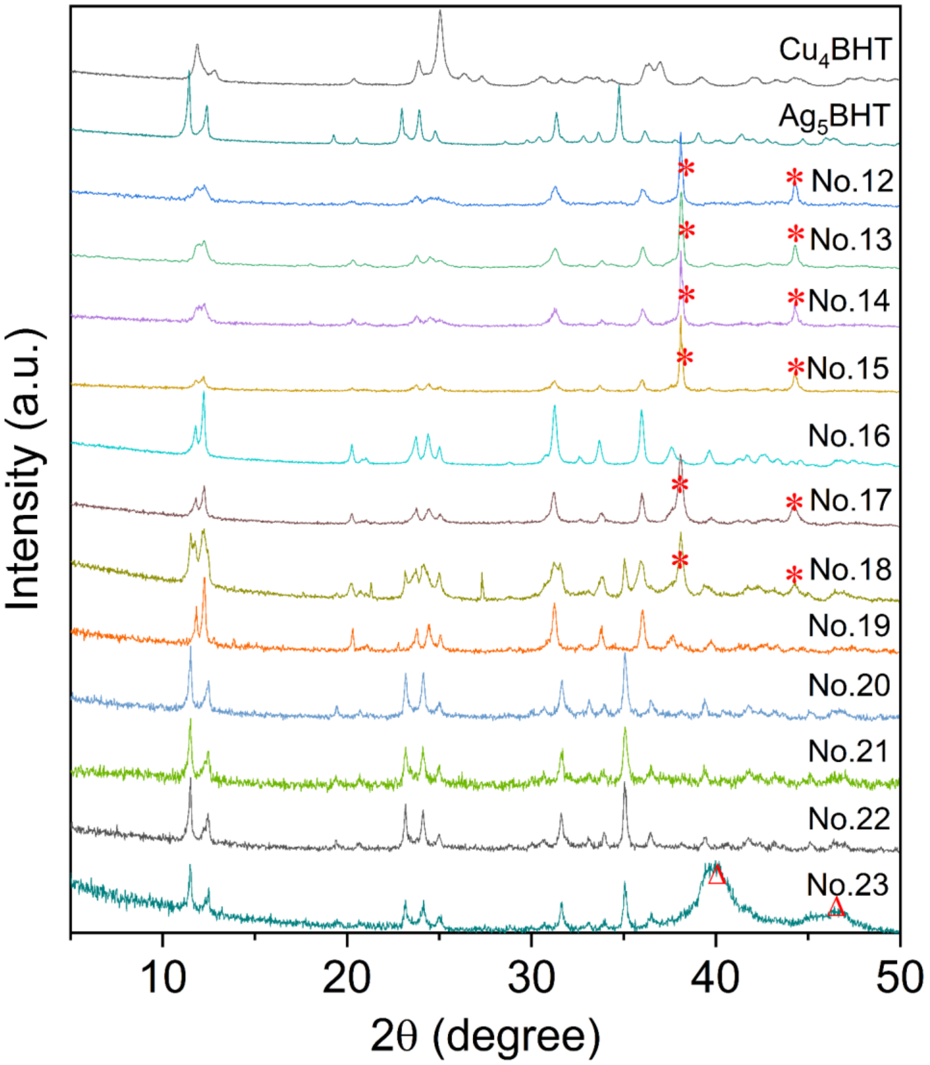

Supplementary Figure 2. PXRD patterns of the products synthesized under different conditions (Supplementary Table 2). The peaks at $2\theta = 38.1°$ and 44.3° (marked by the asterisks) can be indexed to the Ag FCC phase. Two typical peaks at 2θ = 40° and 47° (marked by the triangles) can be assigned to (111) and (200) reflections of the Pd FCC phase[11]. Source data are provided as a Source Data file.

Although these two parent OMCs (Cu_4BHT and Ag_5BHT) have similar building blocks and coordination geometries, the transmetalation results are significantly different. For instance, whether adjusting the concentrations and types of silver salts or the reaction temperature, the metal replacement reaction of Cu_4BHT with silver ion always produces a mixture containing Ag metal, as evidenced by the PXRD characterizations (No. 12-15). Similarly, the reaction of monovalent Cu with Ag_5BHT also leads to the formation of Ag metal, and all these mixtures display a significant loss of crystallinity. It can be inferred that Ag ions in the coordinated or free states could be easily reduced to elemental metal by Cu(I) during the transmetalation process, thereby destroying the crystallinity of metathesis products. Besides, other divalent metal ions (e.g., Mn^{2+}, Fe^{2+}, Co^{2+}, Pd^{2+}) (No. 20-23) failed to yield exchange products, probably owing to the mismatched ion radius and poor thiophilicity compared with copper(II)[12].

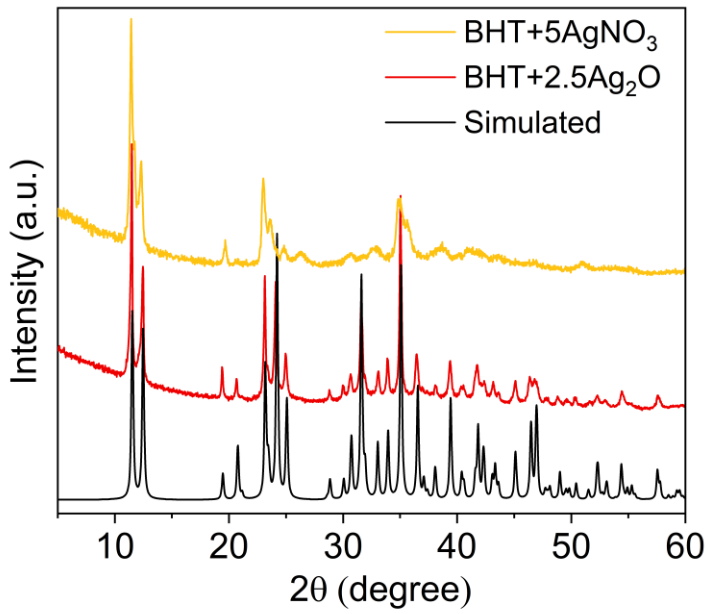

Supplementary Figure 3. The comparison of PXRD patterns of the products synthesized by $AgNO_3$ and Ag_2O. As confirmed by sharp, well-isolated diffraction peaks, the crystallinity of Ag_5BHT with Ag_2O as the reactant is much higher than that of the product obtained with nitrates as the reactant. Source data are provided as a Source Data file.

a b

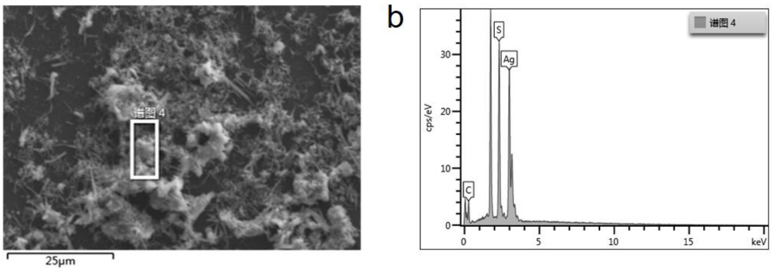

Element	At. NO.	Lines.	Wt%	Wt% Sigma	Atom%
S	16	K-serie	20.03	0.28	21.49
Ag	47	L-serie	59.15	0.64	18.86
			Ag/S atomic ratio = 1: 1.14		

c d

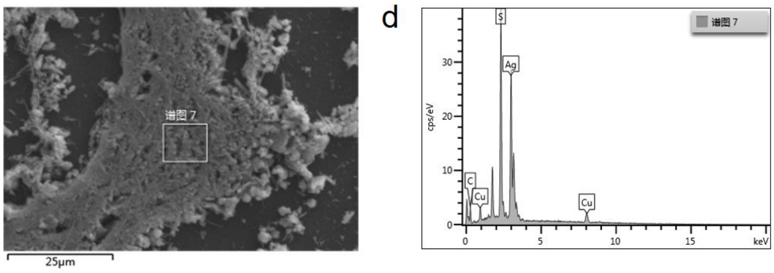

Element	At. NO.	Lines.	Wt%	Wt% Sigma	Atom%
S	16	K-serie	21.93	0.29	25.73
Cu	29	L-serie	7.51	0.41	4.45
Ag	47	L-serie	54.31	0.59	18.94
			Cu/Ag/S atomic ratio = 1: 4.25: 5.78		

e f

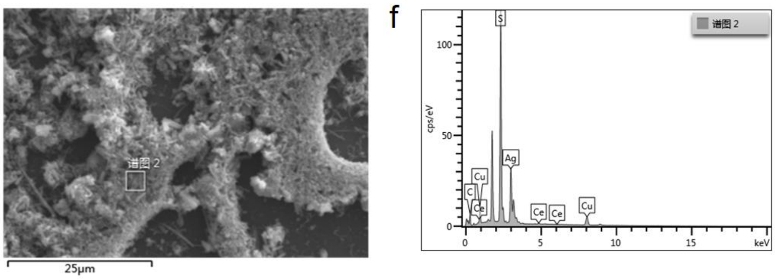

Element	At. NO.	Lines.	Wt%	Wt% Sigma	Atom%
S	16	K-serie	31.39	0.23	26.76
Cu	29	L-serie	10.76	0.19	4.63
Ag	47	L-serie	30.56	0.26	7.74
Ce	58	L-serie	0.59	0.09	0.12
			Cu/Ag/S atomic ratio = 1:1.67:5.78		

Supplementary Figure 4. The Electron Probe Micro-analyzer (EPMA) characterization performed on powder samples of Ag_5BHT (**a,b**), $CuAg_4BHT$ (**c,d**) and $CuAg_2BHT$ (**e,f**). The corresponding element contents are listed in the tables below.

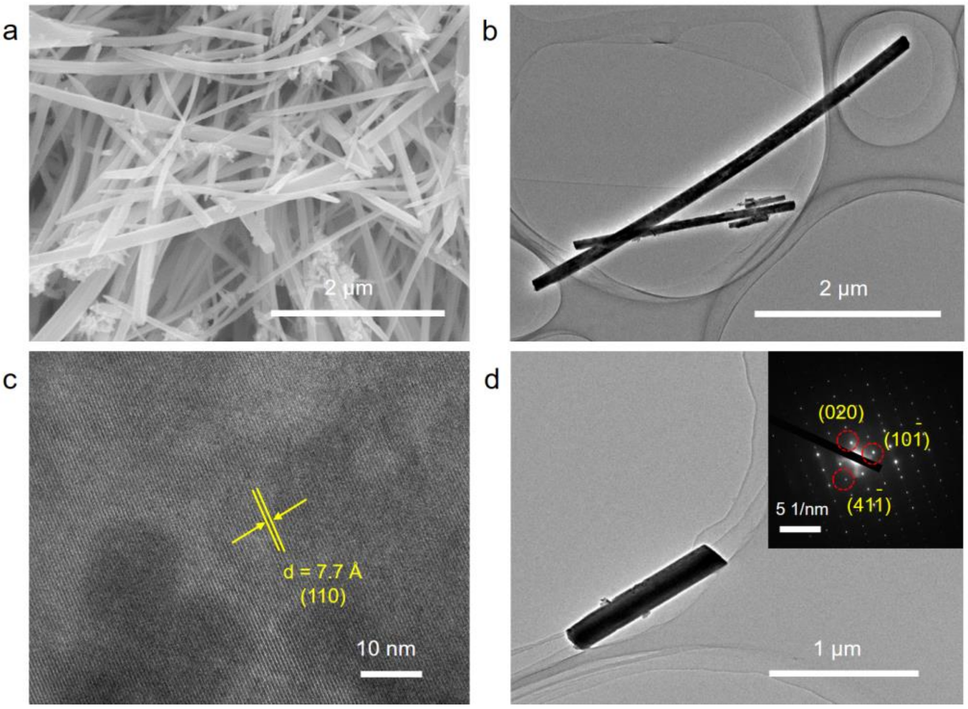

Supplementary Figure 5. The morphology characterization of Ag_5BHT. **a,b,** SEM (**a**) and TEM (**b**) images of Ag_5BHT. **c,** HRTEM image of Ag_5BHT. The clear lattice fringes with the interlayer spacing of 7.7 Å was observed in HRTEM image, corresponding well with the observed diffraction peak at $2\theta = 11.48°$ (Supplementary Fig. 3). **d,** TEM image of Ag_5BHT and its corresponding SAED pattern. All the SEM, TEM and SAED results verified that the Ag_5BHT prepared with Ag_2O as precursors are highly crystalline, which is critically important for the subsequent metal metathesis.

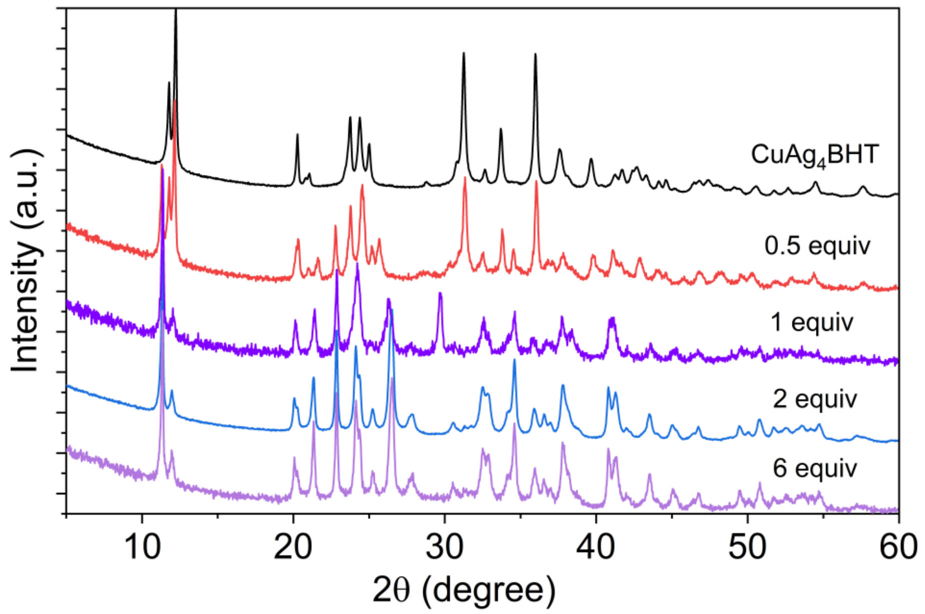

Supplementary Figure 6. PXRD patterns of the oxidized products of $CuAg_4BHT$ under different stoichiometry of CAN. In order to optimize the reaction conditions, different amounts of CAN were added to $CuAg_4BHT$ using acetonitrile as a reaction medium. When the addition of CAN is less than two equivalents, the transition phases or mixed phases can be clearly observed. Source data are provided as a Source Data file.

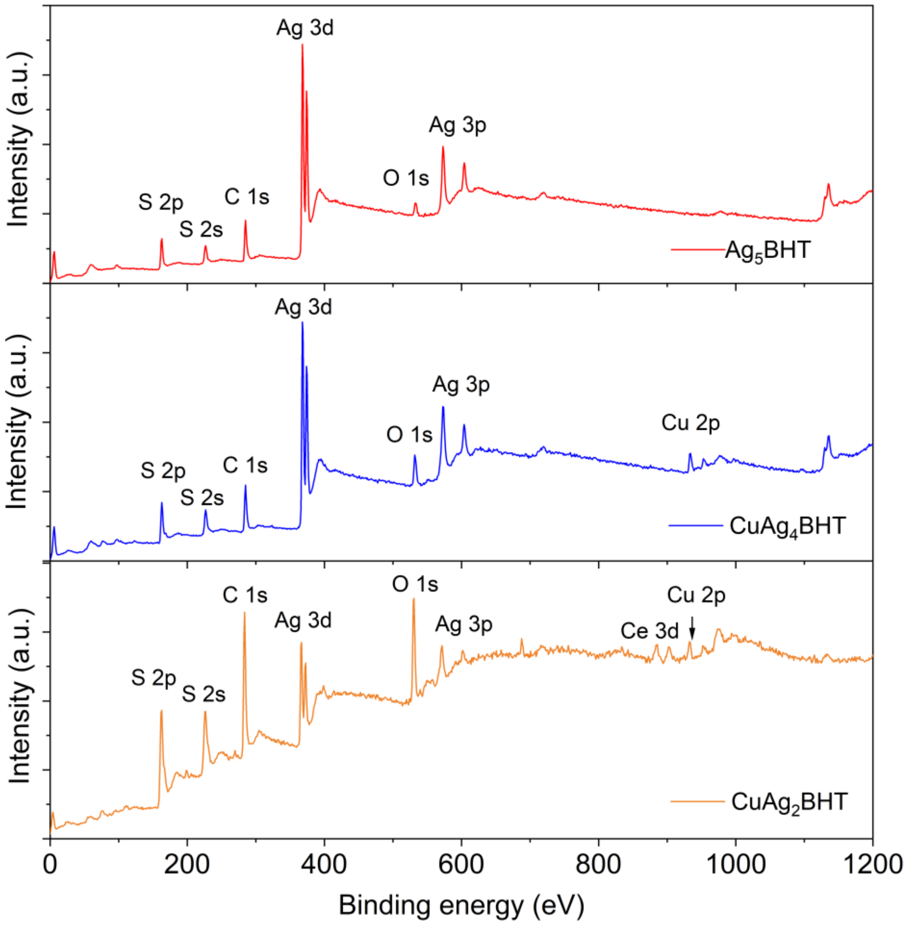

Supplementary Figure 7. The XPS full spectrum of Ag_5BHT and $CuAg_xBHT$. Source data are provided as a Source Data file.

The XPS full spectrum revealed the presence of carbon, sulfur, and silver in $Ag_5C_6S_6$, and the presence of C, S, Cu, and Ag in $CuAg_xC_6S_6$. Besides, the signal belonging to Ce(III) species was detected in $CuAg_2BHT$. Elemental analysis and EPMA results showed that the amount of Ce(0.59%) in this sample was nearly negligible, indicating that Ce^{3+} ions may originate from the adsorption on the surface layer of the product during the oxidation process. The O 1*s* peaks at ~531.8 eV can be ascribed to surface-adsorbed oxygen species (adsorbed from the solvent or from the air atmosphere)[13-15], which is general in the other organic metal chalcogenides[16-20]. As illustrated in Fig. 1, from the 1st generation of Ag_5BHT (coordination assembly), to the 2rd generation of $CuAg_4BHT$ (metal metathesis), and then to the 3rd generation of $CuAg_2BHT$ (oxidation regulation), the deployment of post-synthesis methods gradually increases the defect states in the attained products, which has also been discussed in other bimetallic MOFs[21,22]. These stepwise processes inevitably provide more coordination unsaturated sites for the adsorption of oxygen species and result in a more significant O 1*s* peak.

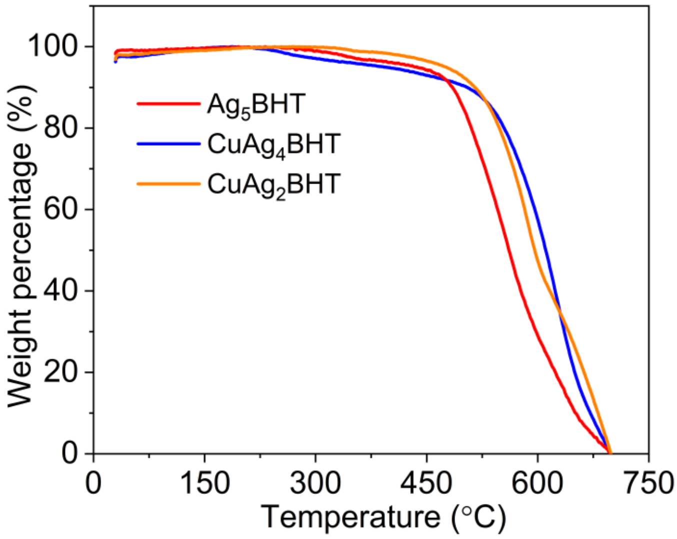

Supplementary Figure 8. Thermogravimetric analysis (TGA) curves for the decomposition of Ag_5BHT and $CuAg_xBHT$. TGA results show the thermal stability up to 300 °C, suggesting that no solvent molecules are contained in the final products. Source data are provided as a Source Data file.

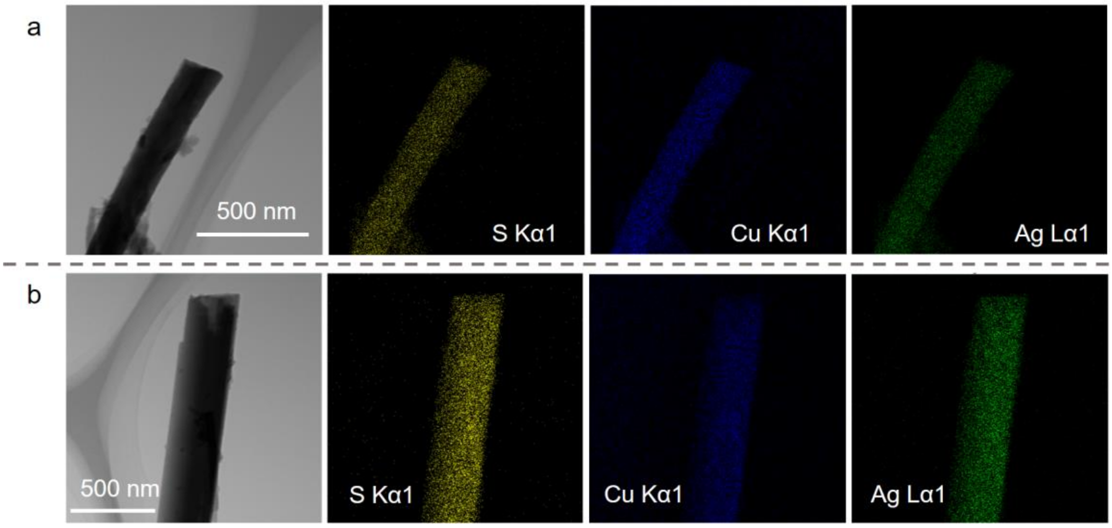

Supplementary Figure 9. TEM images of $CuAg_4BHT$ (**a**) and $CuAg_2BHT$ (**b**) nanorods and the corresponding EDS mapping. The EDS results disclosed the uniform distribution of S, Cu and Ag across the samples, suggesting that the samples prepared herein were not physical mixtures of two monometallic OMCs.

Supplementary Table 3. The comparison of some similar *d* spacings of Ag_5BHT and $CuAg_4BHT$ obtained from PXRD patterns.

Ag_5BHT				$CuAg_4BHT$			
(hkl)	*d* spacing (Å)	(hkl)	*d* spacing (Å)	(hkl)	*d* spacing (Å)	(hkl)	*d* spacing (Å)
(110)	7.6673	($\bar{1}$21)	3.1168	(011)	7.4868	($\bar{1}$13)	3.1033
(200)	7.0898	(130)	2.9708	(002)	7.2219	(121)	2.9567
($\bar{1}$01)	4.2719	(211)	2.9041	(100)	4.2652	(113)	2.9052
(310)	4.1959	($\bar{5}$01)	2.8276	(021)	4.1892	(031)	2.8604
(220)	3.8337	(420)	2.7981	(110)	3.8343	(024)	2.7855
($\bar{2}$11)	3.7937	(510)	2.7079	($\bar{1}$02)	3.7997	(032)	2.7057
($\bar{3}$01)	3.7785	(301)	2.6382	($\bar{1}$11)	3.7697	($\bar{1}$23)	2.6446
(011)	3.6728	(330)	2.5558	(111)	3.6454	(114)	2.5443
(101)	3.5502	($\bar{2}$31)	2.4561	(102)	3.5573	(033)	2.4956
(400)	3.5549	($\bar{5}$21)	2.4027	($\bar{1}$12)	3.4856	(006)	2.4073

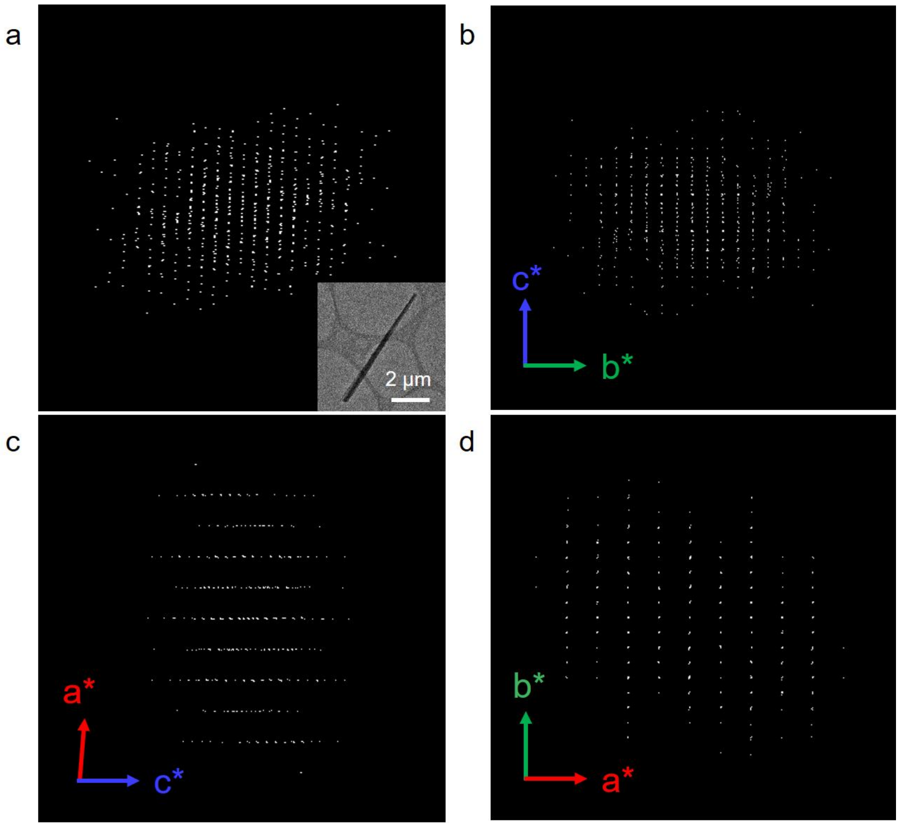

Supplementary Figure 10. 3D reciprocal lattice of $CuAg_4BHT$ reconstructed from RED data. **a,** The 3D view of the diffraction data. The inset is the microcrystal employed to collect the RED data. **b-d,** Diffraction patterns of view along the *a**, *b** and *c** axis, respectively.

Supplementary Table 4. Fractional atomic coordinates and equivalent isotropic displacement parameters for $CuAg_4C_6S_6$ (upper) and $CuAg_2C_6S_6$ (lower).

Atom	*x/a*	*y/b*	*z/c*	Uiso [Å^2]
Ag1	0.229(5)	0.817(2)	0.196(18)	0.056(4)
Ag2	-0.270(5)	0.663(2)	0.301(18)	0.056(4)
Cu1	0.5	1	0	0.056(4)
S3	0.704(19)	0.823(9)	0.099(7)	0.056(4)
S1	0.040(9)	1.005(11)	0.318(2)	0.056(4)
S2	0.316(18)	0.681(9)	0.405(7)	0.056(4)
C1	0.322(5)	0.993(3)	0.415(2)	0.068(4)
C2	0.395(15)	0.858(11)	0.466(16)	0.023
C3	0.378(3)	1.139(12)	0.458(12)	0.023

Atom	*x/a*	*y/b*	*z/c*	Ueq [Å^2]
S1	0.780(16)	0.431(5)	0.297(5)	0.017
S2	0.047(2)	0.202(6)	0.102(4)	0.017
S3	0.306(18)	0.782(6)	0.310(6)	0.017
C1	1.112	-0.188	0.584	0.017
C2	0.986	-0.096	0.680	0.017
C3	0.874	0.093	0.596	0.017
Ag1	0.5	0	0	0.017
Ag2	0.5	0.5	0	0.017
Cu1	0.5	0.5	0.5	0.017

Supplementary Table 5. Elemental analysis results of Ag_5BHT and $CuAg_x$BHT samples.

Formula	C (wt%)		S (wt%)		Cu (wt%)		Ag (wt%)	
	E[a]	T[b]	E[a]	T[b]	E[a]	T[b]	E[a]	T[b]
$Ag_5C_6S_6$	9.12	8.97	23.32	23.93	/	/	67.43	67.10
$CuAg_4C_6S_6$	9.79	9.49	25.46	25.33	9.20	8.37	55.55	56.81
$CuAg_2C_6S_6$	14.53	13.25	36.16	35.38	10.23	11.69	38.48	39.68

[a] Experimental results. [b] Theoretical values.

Discussions on the purity and stability of three OMCs studied:

All of these OMCs have high phase purity. Firstly, the diffraction patterns of three samples were indexed using N-TREOR09 program integrated in EXPO2014 package based on the first 20 intensive peaks, which gave a credible unit cell with the final Rietveld refinement converging with R_p = 3.45%, 3.57%, 2.21% for Ag_5BHT, $CuAg_4$BHT and $CuAg_2$BHT, respectively. As shown in Fig. 2a,b and Supplementary Fig. 3, no peaks belonging to crystalline impurities could be observed, and the calculated results are in good agreement with the experimentally observed PXRD data, verifying the phase purity of Ag_5BHT and $CuAg_x$BHT samples. Besides, EPMA characterizations provided atomic ratios of Cu: Ag: S approximately 1: 4.25: 5.78 for $CuAg_4$BHT and 1: 1.67: 5.78 for $CuAg_2$BHT (Supplementary Fig. 4), which agrees well with the expected $CuAg_x$BHT formulas. In addition, in order to further check the validity of the obtained formulas, we had performed elemental analysis by a combination of ICP-OES and C, H, N, S combustion method. The ICP-OES and elemental analysis results of $CuAg_x$BHT powder samples are highly consistent with the formulas obtained based on crystal structure analysis and further confirmed the purity unambiguously.

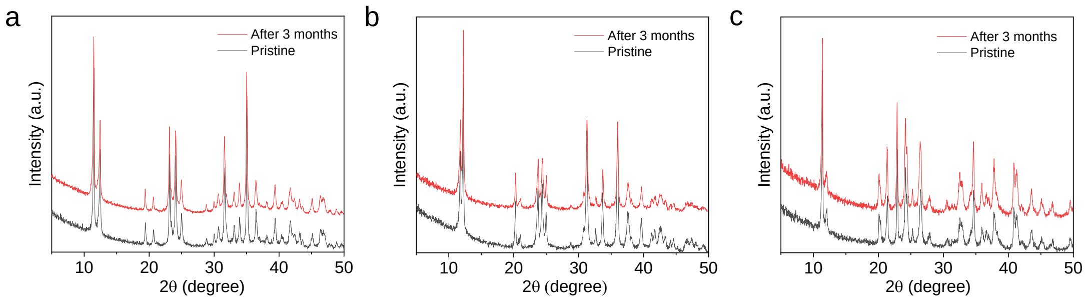

Supplementary Figure 11. The comparison of PXRD patterns of Ag_5BHT (**a**), $CuAg_4$BHT (**b**), $CuAg_2$BHT (**c**) samples before and after three months of air exposure. It was found that no significant changes in the PXRD patterns were observed after they were exposed to ambient air for 3 months. Therefore, these products were believed to have good chemical stability. Source data are provided as a Source Data file.

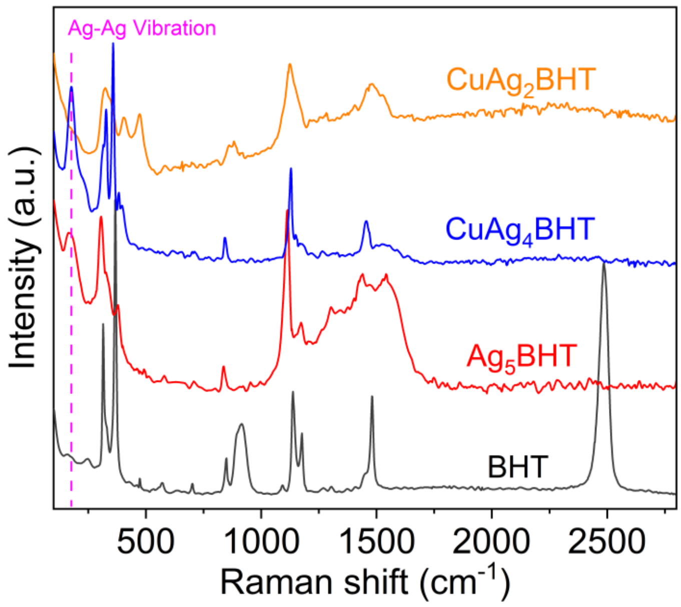

Supplementary Figure 12. Raman spectrum of BHT, Ag_5BHT and $CuAg_xBHT$. Source data are provided as a Source Data file.

The strong Raman signal at ~2490 cm^{-1} attributable to the S-H stretching vibration of BHT vanished in Ag_5BHT, indicating the successful coordination reaction between the -SH groups and Ag ions. The vibration signal of the benzene units at 1400~1590 cm^{-1} can be observed in all OMCs. The symmetric stretching vibration signals of [MS_4] appear at ~305 cm^{-1} for Ag_5BHT and ~330 cm^{-1} for $CuAg_xBHT$. As shown by pink dashed line, both Ag_5BHT and $CuAg_4BHT$ have a characteristic Ag-Ag bond vibration signal at 178 cm^{-1}.

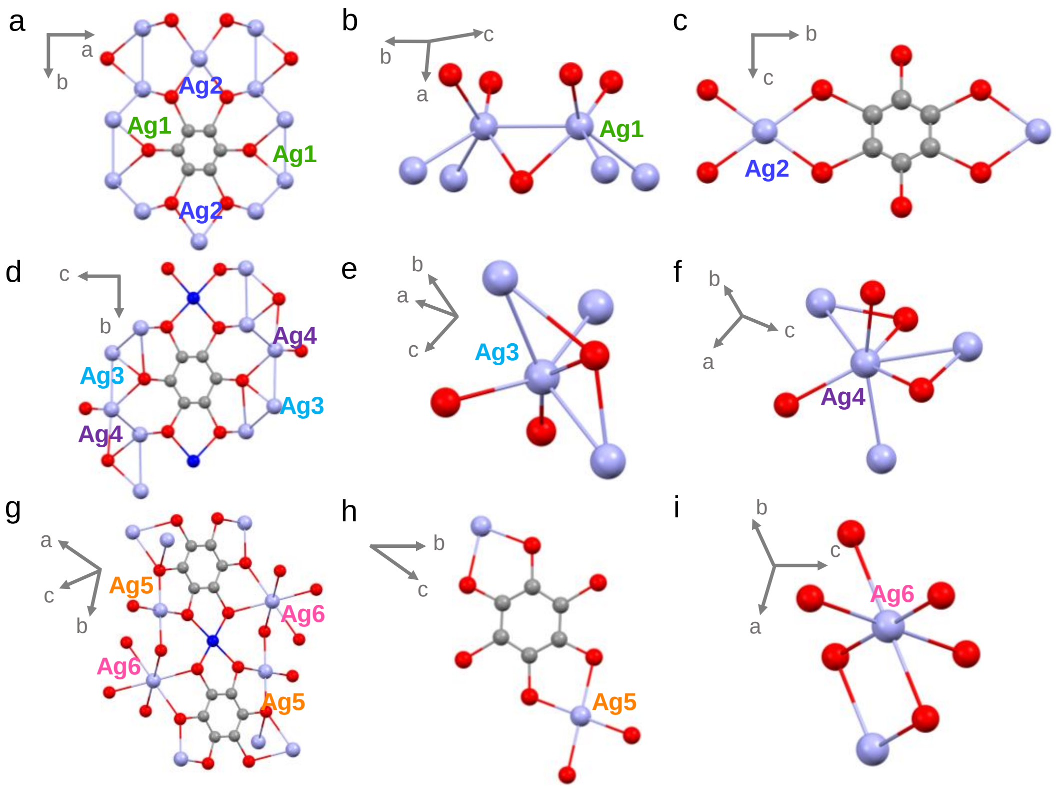

Supplementary Figure 13. Illustration of different coordination modes of Ag atoms in Ag_5BHT (**a-c**), $CuAg_4BHT$ (**d-f**) and $CuAg_2BHT$ (**g-i**).

As shown in supplementary Fig. 13, after metal metathesis, the symmetry-identical Ag atoms (denoted as Ag1) with distorted octahedron geometry in Ag_5BHT change to two symmetry-independent ones (denoted as Ag3 and Ag4), where Ag4 is attached to four S atoms and three Ag3 atoms, displaying a distorted decahedral configuration. It can be seen that two Ag atoms with different coordination geometries exist in these OMCs, which is consistent with the asymmetry signals found in Ag 3*d* XPS spectrum (Supplementary Fig. 14). In these OMCs, Ag atoms exhibit a variety of coordination geometries. Of these, Ag1, Ag3 and Ag6 atoms are six-coordinated, Ag2 and Ag5 atoms are tetra-coordinated, and only Ag4 atoms are seven-coordinated. It is worth noting that square-planar and seven-coordinated silver are generally considered to be rare in all reported silver complexes[23].

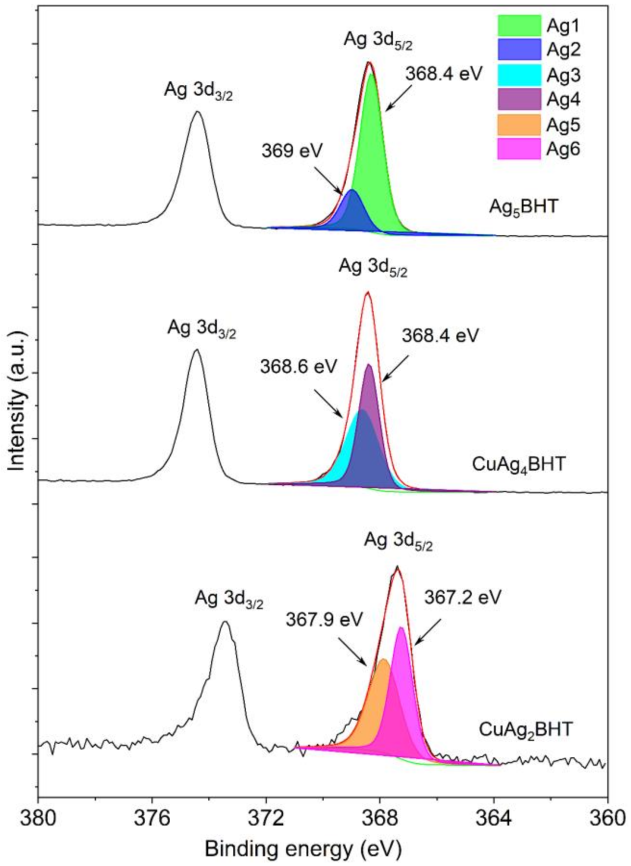

Supplementary Figure 14. XPS spectrum of Ag_5BHT and $CuAg_xBHT$ focusing on Ag 3*d* region. Deconvoluted high-resolution spectrum of the Ag $3d_{5/2}$ in Ag_5BHT and $CuAg_xBHT$ exhibited notable binding energy (B.E.) differences. This difference in B.E. can be attributed to the divergent coordination environments, which are consistent with the respective structural models in Supplementary Fig. 13. Source data are provided as a Source Data file.

Supplementary Table 6. Conductivity values of Ag_5BHT and $CuAg_xBHT$ at 300 K.

Sample name	Average conductivity (S cm^{-1})	Conductivity values (S cm^{-1})		
		Sample 1	Sample 2	Sample 3
Ag_5BHT	10.9	11.3	10.9	10.7
$CuAg_4BHT$	3.03	3.22	2.84	3.04
$CuAg_2BHT$	0.13	0.21	0.11	0.09

Supplementary Table 7. The comparison of the conductivity values of other OMCs at ambient temperature.

Ligands	OMCs	Conductivity (compressed pellets) (S cm^{-1})	References
BHT	**Ag_5BHT**	**10.9**	**This work**
	**$CuAg_4BHT$**	**3.03**	
	**$CuAg_2BHT$**	**0.13**	
	Pb_3BHT	2×10^{-6}	[ref.[24]]
	Ni_3BHT_2	0.15	[ref.[25]]
	Pd-BHT	2.8×10^{-2}	[ref.[26]]
	Au-BHT	1.1×10^{-4}	[ref.[27]]
	Ag_3BHT	39	[ref.[20]]
	Cu_3BHT	280	[ref.[28]]
	Mn_3BHT	0.39	[ref.[29]]
	Ni_3BHT	5	[ref.[18]]
PTC	Fe_3PTC	10	[ref.[30]]
	Ni_3PTC	9	[ref.[31]]
	Co_3PTC	45	[ref.[32]]
THT	Pt_3THT_2	3.86×10^{-6}	[ref.[33]]
	Co_3THT_2	1.4×10^{-3}	[ref.[34]]
BHS	Cu_3BHS	110	[ref.[35]]
HSTP	$Co_3(HSTP)_2$	1×10^{-6}	[ref.[36]]

PTC = perthiolatedcoronene; THT = 2,3,6,7,10,11-triphenylnenehexathiol;
BHS = benzenehexaselenolate; HSTP = 2,3,6,7,10,11-triphenylenehexaselenol.

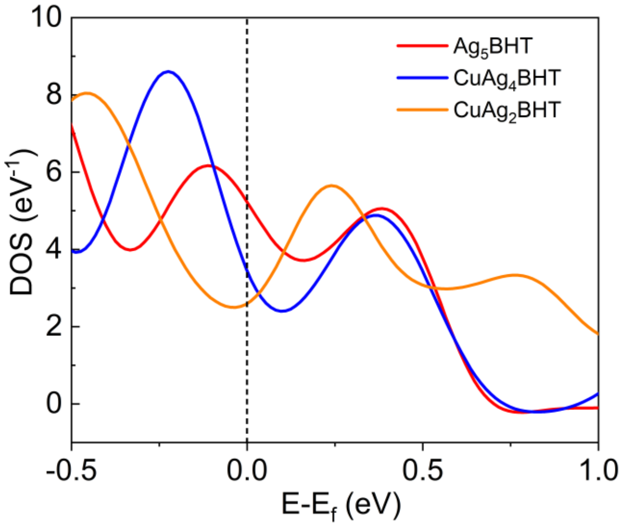

Supplementary Figure 15. The DOS of Ag_5BHT and $CuAg_xBHTs$ near the Fermi level. Source data are provided as a Source Data file.

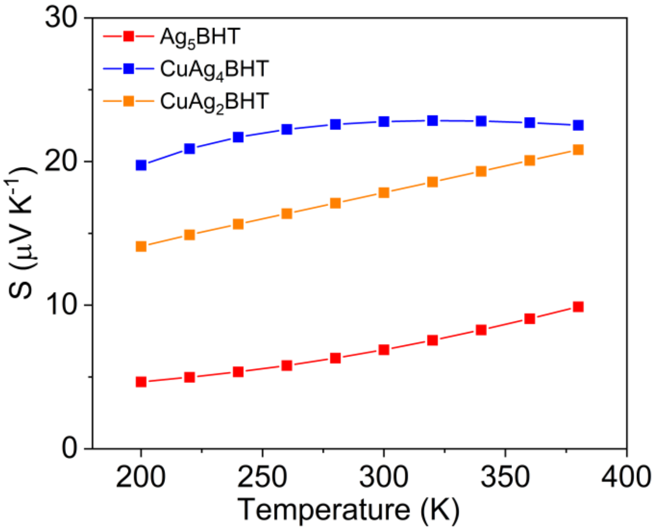

Supplementary Figure 16. Temperature dependence of Seebeck coefficients of Ag_5BHT and $CuAg_xBHT$ simulated via Boltzmann transport equation using Boltztrap2 code. Source data are provided as a Source Data file.

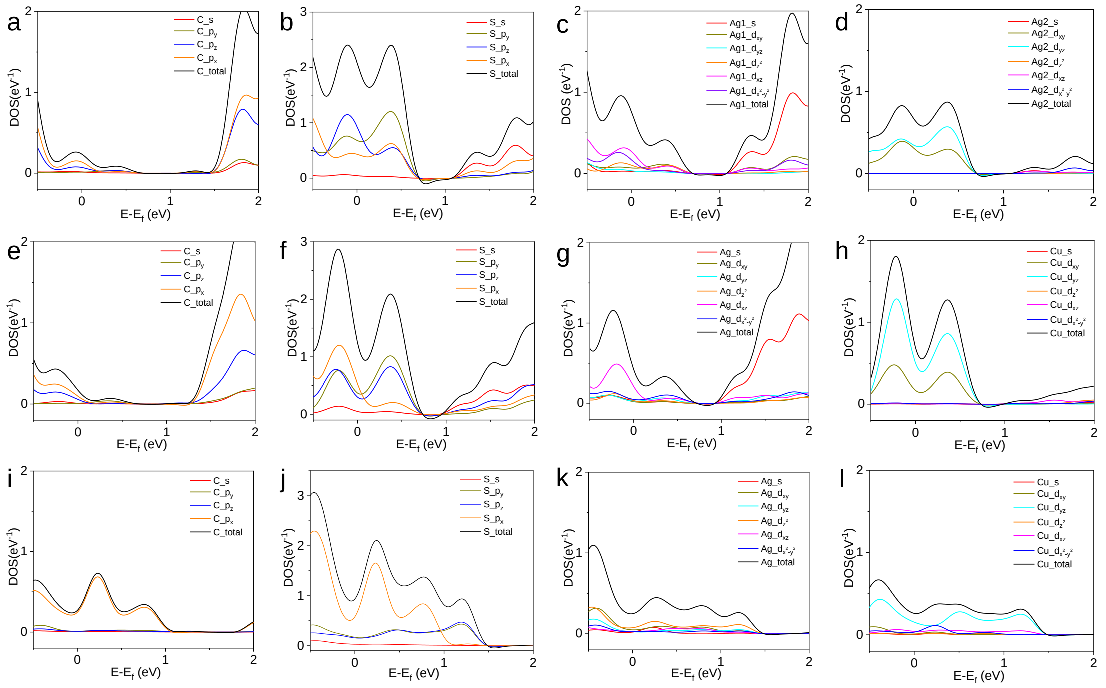

Supplementary Figure 17. Partial density of states (PDOS) of Ag_5BHT (**a-d**), $CuAg_4$BHT (**e-h**) and $CuAg_2$BHT (**i-l**). Source data are provided as a Source Data file.

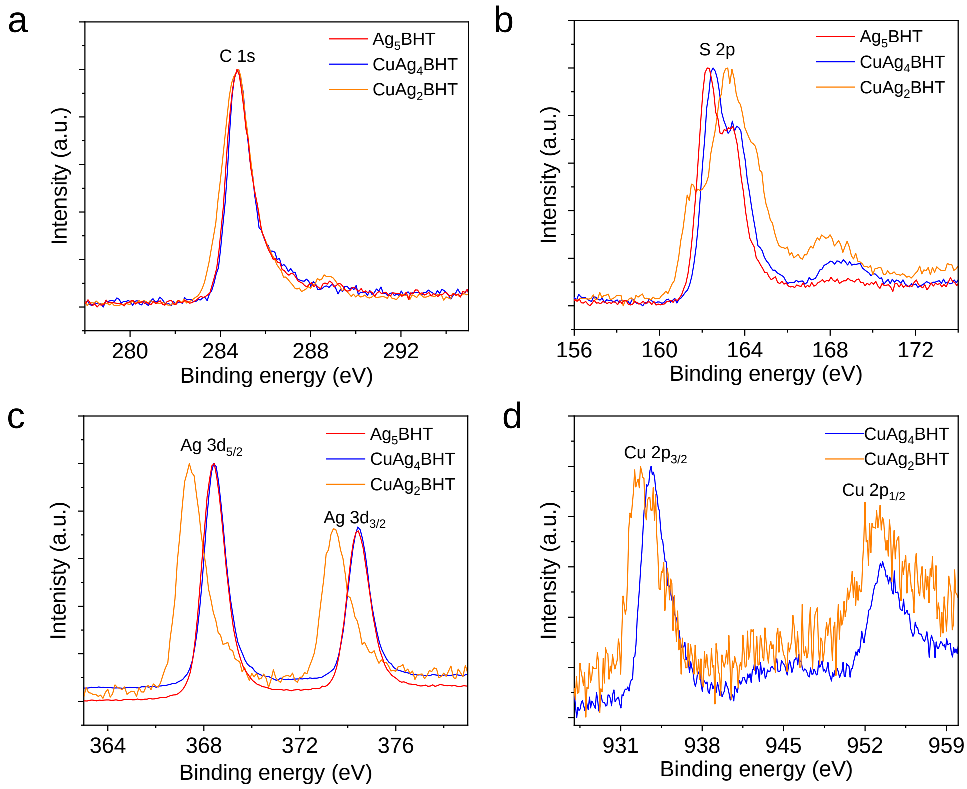

Supplementary Figure 18. The comparison of high resolution XPS spectrum of Ag_5BHT and $CuAg_x$BHTs. The comparison of C 1*s* (**a**), S 2*p* (**b**), Ag 3*d* (**c**) and Cu 2*p* (**d**) region of Ag_5BHT and $CuAg_x$BHTs, respectively. Source data are provided as a Source Data file.

In Cu 2*p* spectrum, a set of peaks at 933.9 and 953.5 eV are assigned to Cu $2p_{3/2}$ and Cu $2p_{1/2}$,

respectively, suggesting the presence of one type of Cu (II) in the $CuAg_4BHT$[37], which was also verified by the magnetic susceptibility measurement. Considering the charge neutrality of the framework, the chemical state of the ligand moiety can be inferred to be -6. Compared to that of Ag_5BHT, a metal metathesis induced charge redistribution could be found in this OMC. Different from $CuAg_4BHT$, the Cu 2*p* region of $CuAg_2BHT$ shows two signals with lower binding energies of 932.6 and 952.9 eV (Supplementary Fig. 18d), which correspond to Cu $2p_{3/2}$ and Cu $2p_{1/2}$, respectively, indicating the presence of Cu (I) in the $CuAg_2BHT$[17]. Thus, the oxidation state of the C_6S_6 moiety can be proposed as -3, which clearly indicates that the chemical state changes occurred not only on the metal nodes but also on the ligand moieties during the chemical transformation process.

Compared to $CuAg_4BHT$, both the C 1*s* and S 2*p* XPS spectrum in $CuAg_2BHT$ have the noticeable sidebands at 288.8 eV and 167.9 eV, respectively (Supplementary Fig. 18a,b). These signals can be attributed to the changes of the oxidation states[32,38] caused by CAN. Besides, the dominant S 2*p* peak in XPS spectrum exhibits an obvious shift (~0.6 eV) towards higher binding energy, indicating that the oxidation process is ligand-centered. The discernable S 2*p* peak at ~167.9 eV in $CuAg_xBHTs$ indicates that adsorbed oxygen strongly interacts with sulfur forming the sulfate or sulfite-like species, which can often be found in the other dithiolene-type compounds[16,31,39-41]. Nonetheless, because XPS is a surface analysis technique with a detection depth of about 3-10 nm, it is clear that there is some oxygen species presenting on the surface rather than the bulk samples, as also evidenced by the phase purity analysis discussed in the main text. Interestingly, the Cu 2*p* spectrum in $CuAg_2BHT$ shifts towards a lower binding energy, although both structures of $CuAg_4BHT$ and $CuAg_2BHT$ have [CuS_4] units. It is unambiguous that these shifts are not caused by metal-centered oxidation, but are associated with the electron transfer or delocalization from the ligand to the metal moieties, thereby forming a Cu(I)-based OMC. Furthermore, Ag atoms in $CuAg_2BHT$ are capped by more S atoms from ligands (Supplementary Fig. 13), which explains why the Ag 3*d* peak of $CuAg_2BHT$ also possesses a lower binding energy than that of $CuAg_4BHT$.

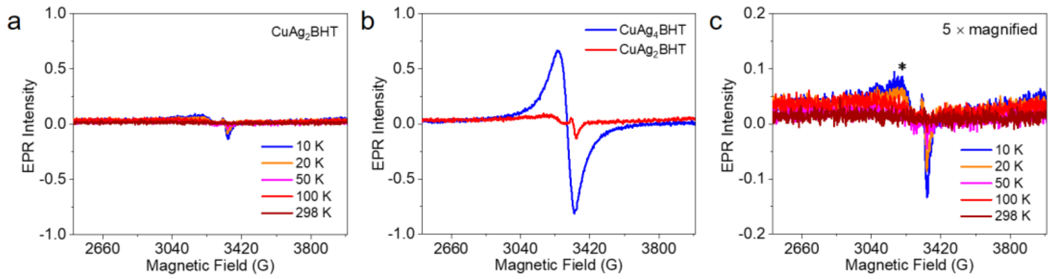

Supplementary Figure 19. Temperature variable EPR spectrum of $CuAg_2BHT$ (**a**). The comparison of EPR spectrum of $CuAg_4BHT$ and $CuAg_2BHT$ at 10 K (**b**). (**c**) shows the same spectra as (**a**), but with signal intensity scaled up by a factor of 5. The signal labelled by the asterisk is from cavity background in the resonator. Source data are provided as a Source Data file.

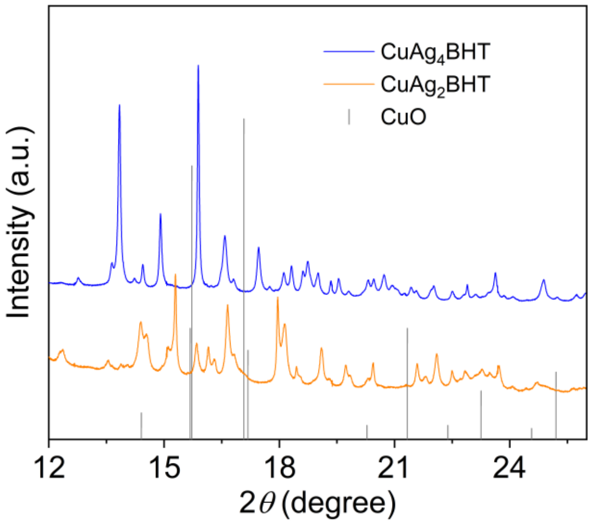

Supplementary Figure 20. The comparison of synchrotron PXRD data of $[CuAg_x(C_6S_6)]_n$ samples and copper oxide. Source data are provided as a Source Data file.

Exclusion of the possible influence of oxides impurities on the magnetic properties of $CuAg_xBHT$:

Firstly, the diffraction data of $[CuAg_x(C_6S_6)]_n$ were collected at synchrotron radiation facility (λ = 0.69003 Å), which has a high sensitivity for the identification of copper oxide impurities. The comparison of synchrotron PXRD data of $[CuAg_x(C_6S_6)]_n$ and CuO was presented in Supplementary Fig. 20, no diffraction peaks associated with CuO can be detected in these products.

Secondly, EPR is a powerful technique for detecting these magnetic impurities. According to J.B. Goodenough's study, the Néel temperature (T_N) of CuO is 230 K (ref.[42]), and the T_N of Cu_4O_3 is about 40 K (ref.[43]). So, if the magnetic properties originate from the copper oxides, the EPR signal will disappear when the temperature drops below these characteristic temperatures. We conducted temperature variable EPR spectrum measurements and found that no such phenomenon was observed in these two samples (Fig. 6a and Supplementary Fig. 19).

Furthermore, the spin orbit coupling of Cu *d* electrons is larger than that of S or O *p* electrons, so the linewidth ($\triangle H_{pp}$) of Cu(II) signal is much wider than that of sulfur-oxide radicals. As shown in Fig. 6a, the EPR signal with *g* value of 2.053 and $\triangle H_{pp}$ of 92 Gauss was observed in $CuAg_4BHT$, completely different from the narrower $\triangle H_{pp}$ (2~4 Gauss) and the smaller *g* values (~2.0051)[44-46] of sulfur oxide radials. As for $CuAg_2BHT$, the EPR signal intensity is temperature dependent and diminishes at room temperature (Supplementary Fig. 19c), which indicates that it does not originate from an organic radical. This signal was broad ($\triangle H_{pp}$=32 Gauss) and asymmetrical, which can be attributed to the $g_\perp$ part of the anisotropic EPR spectrum of magnetically dilute Cu(II). Thus, it is assigned to a small amount of leftover Cu(II) during the oxidation. The existence of Cu(II) could be confirmed by EPR, but $CuAg_2BHT$ was found to be diamagnetic at room temperature, suggesting that the number of leftover Cu(II) was nearly negligible (<0.1%). The sharp contrast between $CuAg_4BHT$ and $CuAg_2BHT$ indicates that the measured magnetic properties does not originate from extrinsic oxides, further verifying the dominant source of magnetic properties of $CuAg_4BHT$.

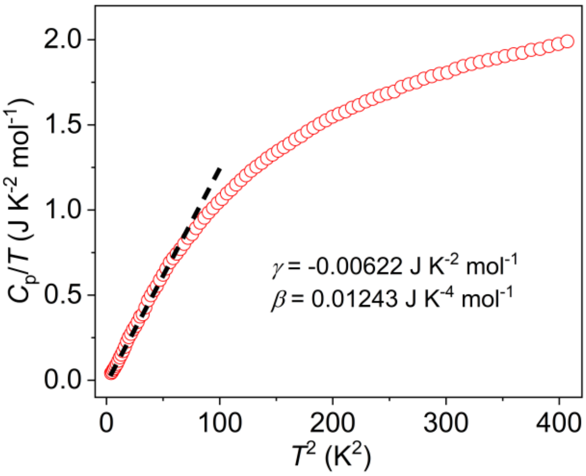

Supplementary Figure 21. C_p/T-T^2 plots of Ag_5BHT in the temperature range of 2-20 K. The dashed line represents a fit using the equation, $C_p = \gamma T + \beta T^3$. Source data are provided as a Source Data file.

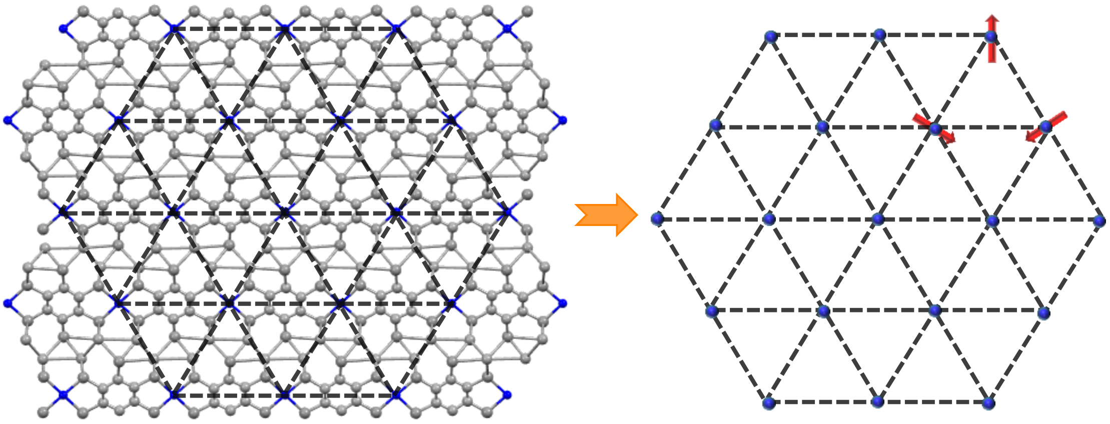

Supplementary Figure 22. Schematic representation of the triangular spin lattice in $CuAg_4$BHT. Blue spheres denote magnetic ions, red arrows indicate the possible direction of spins and black dashed lines indicate the shape of the lattice.

Supplementary Table 8. The high-symmetry K-points of Ag_5BHT, $CuAg_4BHT$ and $CuAg_2BHT$ in the first Brillouin zone.

	Ag_5BHT		
Label	k1	k2	k3
Γ	0.000	0.000	0.000
C	0.353	0.353	0.000
C_2	-0.353	0.647	0.000
Y_2	-0.500	0.500	0.000
M_2	-0.500	0.500	0.500
D	-0.329	0.671	0.500
D_2	0.329	0.329	0.500
A	0.000	0.000	0.500
L_2	0.000	0.500	0.500
V_2	0.000	0.500	0.000

	$CuAg_4BHT$		
Label	k1	k2	k3
Γ	0.000	0.000	0.000
Z	0.000	0.500	0.000
D	0.000	0.500	0.500
B	0.000	0.000	0.500
A	-0.500	0.000	0.500
E	-0.500	0.500	0.500
C_2	-0.500	0.500	0.000
Y_2	-0.500	0.000	0.000

	$CuAg_2BHT$		
Label	k1	k2	k3
Γ	0.000	0.000	0.000
X	0.500	0.000	0.000
Y	0.000	0.500	0.000
Z	0.000	0.000	0.500
R_2	-0.500	-0.500	0.500
T_2	0.000	-0.500	0.500
U_2	-0.500	0.000	0.500
V_2	0.500	-0.500	0.000

Supplementary references

1. Yip, H. K., Schier, A., Riede, J., Schmidbaur, H. Benzenehexathiol as a template rim for a golden wheel: synthesis and structure of [{CSAu(PPh_3)}$_6$]. *Dalton Trans.*, 2333-2334 (1994).
2. Jin, Y. et al. Facile synthesis, precise species control and chemical transformation of highly conducting organic metal chalcogenides Cu_xBHT (BHT = benzenehexathiol; x = 3, 4, and 5.5). *J. Mater. Chem. C*, **10**, 2711-2717 (2022).
3. Huang, X. et al. Highly conducting neutral coordination polymer with infinite two-dimensional silver-sulfur networks. *J. Am. Chem. Soc.* **140**, 15153-15156 (2018).
4. Palatinus, L., Chapuis, G. SUPERFLIP-a computer program for the solution of crystal structures by charge flipping in arbitrary dimensions. *J. Appl. Crystallogr.* **40**, 786-790 (2007).
5. Altomare, A. et al. EXPO2013: a kit of tools for phasing crystal structures from powder data. *J. Appl. Crystallogr.* **46**, 1231-1235 (2013).
6. Altomare, A. et al. Advances in powder diffraction pattern indexing: N-TREOR09. *J. Appl. Crystallogr.* **42**, 768-775 (2009).
7. Petříček, V., Dušek, M., Palatinus, L. Crystallographic computing system JANA2006: general features. *Z. Krist. – Crystal. Mater.* **229**, 345-352 (2014).
8. Perdew, J. P., Burke, K., Ernzerhof, M. Generalized gradient approximation made simple. *Phys. Rev. Lett.* **77**, 3865-3868 (1996).
9. Klimeš, J., Bowler, D. R., Michaelides, A. Van der Waals density functionals applied to solids. *Phys. Rev. B* **83**, 195131 (2011).
10. Yan, Y., Kang, S.-Z., Mu, J. Preparation of high quality Ag film from Ag nanoparticles. *Appl. Surf. Sci.* **253**, 4677-4679 (2007).
11. Pol, V. G., Grisaru, H., Gedanken, A. Coating noble metal nanocrystals (Ag, Au, Pd, and Pt) on polystyrene spheres via ultrasound irradiation. *Langmuir* **21**, 3635-3640 (2005).
12. Mantella, V., Varandili, S. B., Pankhurst, J. R., Buonsanti, R. Colloidal synthesis of Cu–M–S (M = V, Cr, Mn) nanocrystals by tuning the copper precursor reactivity. *Chem. Mater.* **32**, 9780-9786 (2020).
13. Merino N. A., Barbero B. P., Eloy P. & Cadús L. E. $La_{1-x}Ca_xCoO_3$ perovskite-type oxides: Identification of the surface oxygen species by XPS. *Appl. Surf. Sci.* **253**, 1489-1493 (2006).
14. Zhang M. et al. Effects of the surface adsorbed oxygen species tuned by rare-earth metal doping on dry reforming of methane over Ni/ZrO_2 catalyst. *Appl. Catal. B: Environ.* **264**, 118522 (2020).
15. Tabata K., Hirano Y. & Suzuki E. XPS studies on the oxygen species of $LaMn_{1-x}Cu_xO_{3+\lambda}$. *Appl. Catal. A.* **170**, 245-254 (1998).
16. Dong R. et al. Large-area, free-standing, two-dimensional supramolecular polymer single-layer sheets for highly efficient electrocatalytic hydrogen evolution. *Angew. Chem. Int. Ed.* **54**, 12058-12063 (2015).
17. Huang X. et al. A two-dimensional π-d conjugated coordination polymer with extremely high electrical conductivity and ambipolar transport behavior. *Nat. Commun.* **6**, 7408 (2015).
18. Banda H. et al. High-capacitance pseudocapacitors from Li^+ ion intercalation in nonporous, electrically conductive 2D coordination polymers. *J. Am. Chem. Soc.* **143**, 2285-2292 (2021).
19. Toyoda R. et al. Heterometallic benzenehexathiolato coordination nanosheets: Periodic structure improves crystallinity and electrical conductivity. *Adv. Mater.* **34**, 2106204 (2022).
20. Sun Y. et al. Highly conductive organic–inorganic hybrid silver sulfide with 3D silver–sulfur

networks constructed from benzenehexathiol: Structural topology regulation via ligand oxidation. *Inorg. Chem.* **61**, 5060-5066 (2022).
21. Chen L., Wang H.-F., Li C. & Xu Q. Bimetallic metal–organic frameworks and their derivatives. *Chem. Sci.* **11**, 5369-5403 (2020).
22. Raza N., Kumar T., Singh V. & Kim K.-H. Recent advances in bimetallic metal-organic framework as a potential candidate for supercapacitor electrode material. *Coord. Chem. Rev.* **430**, 213660 (2021).
23. Young, A. G., Hanton, L. R. Square planar silver(I) complexes: A rare but increasingly observed stereochemistry for silver(I). *Coord. Chem. Rev.* **252**, 1346-1386 (2008).
24. Turner, D. L., Vaid, T. P., Stephens, P. W., Stone, K. H., DiPasquale, A. G., Rheingold, A. L. Semiconducting lead-sulfur-organic network solids. *J. Am. Chem. Soc.* **130**, 14-15 (2008).
25. Kambe, T. et al. π-conjugated nickel bis(dithiolene) complex nanosheet. *J. Am. Chem. Soc.* **135**, 2462-2465 (2013).
26. Pal, T. et al. Interfacial synthesis of electrically conducting palladium bis(dithiolene) complex nanosheet. *Chempluschem* **80**, 1255-1258 (2015).
27. Chen, I. F., Lu, C. F., Su, W. F. Highly conductive 2D metal-organic framework thin film fabricated by liquid-liquid interfacial reaction using one-pot-synthesized benzenehexathiol. *Langmuir* **34**, 15754-15762 (2018).
28. Huang X. et al. Conductive copper benzenehexathiol coordination polymer as a hydrogen evolution catalyst. *ACS Appl. Mater. Interfaces* **9**, 40752-40759 (2017).
29. Murphy R. A. et al. Exchange bias in a layered metal-organic topological spin glass. *ACS Cent. Sci.* **7**, 1317-1326 (2021).
30. Dong R. et al. A coronene-based semiconducting two-dimensional metal-organic framework with ferromagnetic behavior. *Nat. Commun.* **9**, 2637 (2018).
31. Chen Z. et al. Nanorods of a novel highly conductive 2D metal–organic framework based on perthiolated coronene for thermoelectric conversion. *J. Mater. Chem. C* **8**, 8199-8205 (2020).
32. Chen Z. et al. Highly conductive cobalt perthiolated coronene complex for efficient hydrogen evolution. *Chemistry* **26**, 12868-12873 (2020).
33. Cui J. & Xu Z. An electroactive porous network from covalent metal-dithiolene links. *Chem. Commun.* **50**, 3986-3988 (2014).
34. Clough A. J. et al. Metallic conductivity in a two-dimensional cobalt dithiolene metal-organic framework. *J. Am. Chem. Soc.* **139**, 10863-10867 (2017).
35. Cui Y. et al. $[Cu_3(C_6Se_6)]_n$: The first highly conductive 2D π-d conjugated coordination polymer based on benzenehexaselenolate. *Adv. Sci.* **6**, 1802235 (2019).
36. Cui Y. et al. Synthetic route to a triphenylenehexaselenol-based metal organic framework with semi-conductive and glassy magnetic properties. *iScience* **23**, 100812 (2020).
37. Mendecki L. et al. Porous scaffolds for electrochemically controlled reversible capture and release of ethylene. *J. Am. Chem. Soc.* **139**, 17229-17232 (2017).
38. Li Y. et al. Coordination assembly of 2D ordered organic metal chalcogenides with widely tunable electronic band gaps. *Nat. Commun.* **11**, 261 (2020).
39. Dong R. et al. High-mobility band-like charge transport in a semiconducting two-dimensional metal-organic framework. *Nat. Mater.* **17**, 1027-1032 (2018).
40. Clough A. J. et al. Room temperature metallic conductivity in a metal-organic framework induced by oxidation. *J. Am. Chem. Soc.* **141**, 16323-16330 (2019).

41. Wang Y. C. et al. Two-dimensional bis(dithiolene)iron(II) self-powered UV photodetectors with ultrahigh air stability. *Adv. Sci.* **8**, 2100564 (2021).
42. Goodenough J. B. *Magnetism and the chemical bond*. Hassell Street Press (1963).
43. Pinsard-Gaudart L. et al. Magnetic properties of paramelaconite Cu_4O_3: A pyrochlore lattice with S= 1/2. *Phys. Rev. B* **69**, 104408 (2004).
44. Rinker R. et al. The presence of the SO_2 radical ion in aqueous solutions of sodium dithionite. *J. Phys. Chem.* **63**, 302-302 (1959).
45. Salagram M. et al. ESR characterisation of SO_3^- and SO_4^- radicals in X-irradiated kainite ($KMgClSO_4 \cdot 3H_2O$). *Spectrochimica Acta Part A: Molecular Spectroscopy* **50**, 1309-1315 (1994).
46. Schoonheydt R. & Lunsford J. H. Electron paramagnetic resonance of SO_2-on magnesium oxide. *J. Phys. Chem.* **76**, 323-328 (1972).
